# Can allelopathic potentialities of Mediterranean plant species reduce the spread of invasive plant species, *Acacia dealbata* and *Ailanthus altissima*?

**DOI:** 10.1002/ece3.11499

**Published:** 2024-06-25

**Authors:** Solène Brasseur, Mathieu Santonja, Catherine Rébufa, Laurence Affre, Sylvie Dupouyet, Estelle Dumas, Thierry Tatoni, Anne‐Marie Farnet Da Silva, Anne Bousquet‐Mélou

**Affiliations:** ^1^ Aix Marseille Univ, CNRS, Avignon Univ, IRD, IMBE Marseille France

**Keywords:** allelopathy, invasive alien plant species management, mesocosm experiment, seed germination, seedling growth

## Abstract

Beyond ecological and health impacts, invasive alien plant species can generate indirect and direct costs, notably through reduced agricultural yields, restoration, and management of the invaded environment. *Acacia dealbata* and *Ailanthus altissima* are invasive plant species that cause particularly significant damage to the railway network in the Mediterranean area. The allelopathic properties of Mediterranean plant species could be used as nature‐based solutions to slow down the spread of such invasive plant species along railway borders. In this context, a mesocosm experiment was set‐up: (i) to test the potential allelopathic effects of *Cistus ladanifer*, *Cistus albidus*, and *Cotinus coggygria* leaf aqueous extracts on seed germination and seedling growth of *A. dealbata* and *A. altissima*; (ii) to evaluate whether these effects depend on the extract dose; and finally, (iii) to estimate whether these effects are modified by soil amendment. Leaf aqueous extracts of the three native plant species showed negative effects on both seed germination and seedling growth of the two invasive species. Our results show that the presence of allelochemicals induces a delay in seed germination (*e.g.*, *A. dealbata* germination lasted up to 269% longer in the presence of high‐dose leaf aqueous extracts of *C. coggygria*), which can lead to a decrease in individual recruitment. They also highlight a decrease in seedling growth (*e.g.*, high‐dose *C. coggygria* leaf aqueous extracts induced a 26% decrease in *A. dealbata* radicle growth), which can alter the competitiveness of invasive species for resource access. Our results also highlight that compost addition limits the inhibitory effect of native Mediterranean plants on the germination of invasive alien plants, suggesting that soil organic matter content can counteract allelopathic effects on invasive alien plants. Thus, our findings revealed that the allelopathic potential of certain Mediterranean plant species could be a useful tool to manage invasive plant species.

## Graphical abstract

In this study, we provide clear evidence that the leaf aqueous extracts of three Mediterranean native species (*Cistus albidus*, *Cistus ladanifer* and *Cotinus coggygria*) exhibit negative allelopathic effects on seed germination and seedling growth of the two invasive plant species (*Acacia dealbata* and *Ailanthus altissima*). These allelopathic effects increased with dose extract but are greatly reduced and even canceled in the presence of soil amendment.

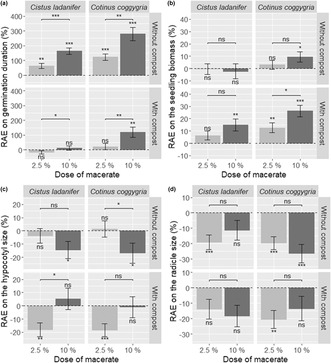

## 1 INTRODUCTION

Human activities are causing a significant redistribution of species on a global scale (Mack et al., 2000). This redistribution has generated an increase in invasive alien species (IAS) since 1970 (Butchart et al., 2010), and there is no sign of a slowdown in species introductions (Seebens et al., 2017). IAS are considered to be one of the main factors affecting native species assemblages and associated ecosystem processes (Cronk & Fuller, 1995; Fried, 2007; Sarat et al., 2015). Indeed, nearly one‐third of threatened terrestrial species and half of known extinctions are due to IAS (IUCN, 2015b). Furthermore, IAS imposes costs not only through reduced agricultural yields, the management of species in the field, or the restoration of invaded environments, but also through human health (IUCN, 2015a). Species invasion processes depend on the characteristics of the invaded environment and those of the introduced species (Fried, 2007). For example, the environment may often be more vulnerable to invasion in the context of vacant ecological niches (resources not fully used by native species), especially in a disturbed environment (Mack et al., 2000), such as burned or flooded habitats, agricultural ecosystems, urbanized habitats, or transportation infrastructures (i.e., roads or railways). In this particular context, the relationship between plants and soils is a subtle equilibrium that conditions the potential spread after introduction/naturalization events. Thus, soil physicochemical properties, land use history, microbial community, and nutrient resources all influence the establishment of invasive alien plant species, which in turn can alter soil chemical properties (Brett Mattingly & Orrock, 2013; Lankau, 2010; Li et al., 2017). For example, Davis et al. (2000) found that a plant community becomes more susceptible to plant invasion as the amount of unused soil resources increases, illustrating that soil properties condition the colonization potentialities of invasive alien plants. On the other hand, Souza et al. (2011) reported that the increased availability of nitrogen in the soil directly stimulates the growth of native plant species, thus limiting the access of invasive alien plants to other resources such as light or water. In parallel, intrinsic parameters that favor the invasion of alien plant species include a high reproduction rate, tolerance to environmental factors such as pollutants (Goudard, 2007), a high resource exploitation capacity, or allelopathic abilities (Bais et al., 2003; Callaway & Aschehoug, 2000; Callaway & Ridenour, 2004).

Indeed, allelopathy (i.e. any direct or indirect, positive, or negative effect of one plant—including microorganisms— on another, through biochemical compounds released into the environment, whether the atmosphere or the soil (Molisch, 1937)) may be involved in the plant invasion success as a chemical weapon for invasive plants (Callaway & Aschehoug, 2000; Callaway & Ridenour, 2004). Such interactions play a key role in the germination and growth of plant species (Callaway & Walker, 1997). Allelochemicals, that is, biomolecules with allelopathic effects, such as phenolic compounds, terpenes, or alkaloids, are often synthesized in small amounts by plants (Doré et al., 2004; Freire et al., 2005, 2007; Kunii et al., 1996; Pereira et al., 1996). However, their production seems to be stimulated under stress conditions such as pathogen or herbivore attack, drought, mineral element deficiency, or UV radiation (Reigosa et al., 2000). Allelochemicals can be released into the environment through rainfall, leaf litter decomposition, volatilization, or root exudation (Lalljee & Facknath, 2000; Reigosa et al., 2000). They can induce inhibition and delay of seed germination in the target plant species (Fernandez et al., 2013; Vivanco et al., 2004). Other modifications can be observed, such as inhibition of cell division and elongation or inhibition of protein synthesis, modification of membrane permeability and mineral uptake, modification of photosynthesis and respiration, or even negative interference with growth hormones (Doré et al., 2004; Linhart et al., 2015). The study of the role of allelochemicals within the ecosystem is complex, mainly because of the interactions that may exist between these molecules and the different soil components (Blum, 2006). Indeed, soil biotic and abiotic characteristics are fundamental for the expression of the allelopathic potential of plants (Gallet & Pellissier, 2002). Allelochemicals can be involved in microbial degradation, absorption, or polymerization (Lankau, 2010; Li et al., 2017; Xuan et al., 2005; Zhang & Fu, 2010). Thus, soil properties (pH, solute concentration, cation exchange capacity, and fertility) affect the activity of allelochemicals (Parepa & Bossdorf, 2016). However, these chemical interactions between plants could also be used to their disadvantage to control their development. The sclerophyllous vegetation of many native Mediterranean plant communities is particularly rich in specialized terpenoid and phenolic metabolites. These compounds give this sclerophyllous vegetation a high allelopathic potential and help it cope with the stresses of the Mediterranean climate, such as summer drought and high levels of radiation (Scognamiglio et al., 2013).

*Acacia dealbata* (Fabaceae) is native to Australia and was introduced in Europe as an ornamental plant in the early 1790s, invading local environments in southern France in the 1860s (Lorenzo et al., 2010; Sheppard et al., 2006). This plant is a species in warm and dry environments that grows spontaneously on acidic soils near plantations and benefits from human disturbances. Its rapid colonization disrupts the natural dynamics of native plant formations, acidifies soils, and causes a homogenization of the environment. It spreads mainly by suckering and stump sprouting, two types of vegetative reproduction that are accentuated when individuals are under stress (e.g. root damage, pruning, cutting) (Lorenzo et al., 2010). *Ailanthus altissima* (Simaroubaceae) is native to southern China and was introduced to Europe as an ornamental plant in the 18th century (Kowarik & Säumel, 2007). This plant species proliferates especially in disturbed environments such as old wastelands, roadsides, or along rail banks. It can tolerate variations in temperature, humidity, and light, grow indifferently on various soil types (including poor soils), and tolerate an acidic pH (Miller, 1991). Current management is based on the use of phytosanitary products and the cutting of stump sprouts several times a year. In the Mediterranean region, both *A. dealbata* and *A. altissima* are considered invasive alien plants and impact Mediterranean ecosystems (Lazzaro et al., 2014; Terzi et al., 2021).

This study is part of the REEVES project, whose main objective is to use the allelopathic potentialities of native Mediterranean plant species as a nature‐based solution to control invasive alien plants along rail banks in southern France (Cheng & Cheng, 2015; Chou, 1999). Indeed, allelopathic compounds produced by native plants can be a weapon to control invasive species and strengthen the resistance of native communities (Chen et al., 2017; Christina et al., 2015; Lopes et al., 2018; Zhao et al., 2008). Thus, the establishment of native Mediterranean plant communities could reduce the development of *Acacia dealbata* (Fabaceae) and *Ailanthus altissima* (Simaroubaceae), which are spreading on rail banks in southern France. To support the development of the native species planted, this management method could include soil amendment. In order to select the native Mediterranean plants to be installed, it is necessary to evaluate the efficiency of their inhibitory potential, depending on the soil amendment and the target species. The objective of this study is to evaluate, in mesocosm experiments, the allelopathic potentialities of three native Mediterranean species (*Cistus ladanifer*, *Cistus albidus*, and *Cotinus coggygria*) on the two target invasive alien plants. Mesocosm experiments were conducted (i) to test whether there is an effect of *C. ladanifer*, *C. albidus*, and *C. coggygria* leaf aqueous extracts on seed germination and seedling growth of *A. dealbata* and *A. altissima*; (ii) to evaluate whether these effects depend on the leaf aqueous extract dose; and finally, (iii) to estimate whether these effects are modified by soil amendment (green waste compost). We hypothesized that (i) the leaf aqueous extracts of the three native plant species will inhibit seed germination and seedling growth of the two invasive alien plants; (ii) the inhibitory effects will increase with the extract dose; and (iii) the soil amendment will buffer the negative allelopathic effects.

## 2 MATERIALS AND METHODS

### 2.1 Plant material

#### 2.1.1 Target plant species

The *Acacia dealbata* seeds were supplied by Les Semences du Puy SARL (origin Italy, harvest 2019–2020), while the *Ailanthus altissima* seeds were harvested during the summer of 2020 on the Aix‐Marseille University site on five different individuals (Saint‐Charles campus) (Kowarik & Säumel, 2007; Lorenzo et al., 2010; Miller, 1991; Sheppard et al., 2006).

#### 2.1.2 Source plant species

*Cistus ladanifer*, *Cistus albidus* (Cistaceae), and *Cotinus coggygria* (Anacardiaceae) are Mediterranean plant species that have demonstrated allelopathic potentialities, mainly inhibitory effects, on different target species (mainly herbaceous species) (Chaves & Escudero, 1997; Gavinet et al., 2019; Herranz et al., 2006; Robles et al., 1999). They were selected as source species because these inhibitory effects could help in the management of invasive alien species in the Mediterranean region. Metabolites active in inhibiting the germination and growth of other target species have been identified, including phenolic compounds such as flavonoids (Castells, 2008; Chaves & Escudero, 1997; Hashoum et al., 2017).

For the experimentation with *A. dealbata* as the target species, the source species were *C. ladanifer* and *C. coggygria*, all of which coexist on siliceous soils, while for the experimentation with *A. altissima* as the target species, the source species were *C. albidus* and *C. coggygria*, all of which coexist on calcareous soils.

Green leaves of source plants were collected in the PACA region (at Roquebrune‐Sur‐Argens for *C. ladanifer*, at the massif de l'Étoile for *C. albidus*, and at Pont Mirabeau for *C. coggygria*) from at least five different individuals during spring 2022.

### 2.2 Experimental design

As among the different modes of release of allelochemicals into the environment, Vyvyan (2002) showed that water‐soluble compounds are the most likely to be involved in allelopathic interactions, natural leachates were approached using aqueous leaf extracts of the source species. Aqueous leaf extracts were prepared by soaking 10% dry mass of fresh leaves in water at room temperature in the dark for 24 h (Souto et al., 1994). Aqueous extracts were then filtered through filter paper (Whatman#1®). Two concentrations were tested: a 10% aqueous extract (high dose) and a 2.5% aqueous extract (low dose) obtained by dilution (Dorning & Don, 2006; McEwan et al., 2010). These two concentrations are usually tested in laboratory experiments consisting of allelopathy bioassays that mimic natural conditions (Fernandez et al., 2013; Gavinet et al., 2019; Hashoum et al., 2017, 2021; Wang et al., 2022).

To ensure a satisfactory germination rate, the seeds were subjected to a pretreatment. *Acacia dealbata* seeds were heat‐shocked by soaking them in boiling distilled water for 5‐vs, followed immediately by cold water (Danthu et al., 2003; Lorenzo et al., 2010). They were then placed in distilled water (control) or in aqueous leaf extract of the source species at room temperature for a 48‐h soaking period. As *Ailanthus altissima* seeds do not require heat shock, they were only placed in water (control) or in leaf aqueous extract of the source species for a 48‐h soaking period. Seeds were grown individually in 60‐ml pots (4 cm in diameter and 5 cm in height).

The effect of amendment addition was tested using a composted and an unamended modality. The amendment was a green waste compost provided by SOLEV (https://solev‐paca.com/). The same compost is used by the SNCF Réseau in the REEVES project to manage invasive alien plants along railways. It was dried in an oven at 60°C for 48 h and sieved using a 2 mm sieve. Pots without compost were filled with 25 g of dry neutral substrate (topsoil dried in an oven at 60°C for 48‐h and sieved through a 4 mm sieve). Pots with compost were filled with 20 g of dry topsoil and 5 g of dry compost (1:5 ratio). The effect of the amendment on the contents of organic C and N and the C/N ratios was quantified at the end of the experiments for three samples of each control treatment (i.e., with and without compost). The samples were dried (60°C for 24 h in a drying oven) and analyzed with a FlashEA 1112 Series Thermofisher. The presence of compost increased the N content, leading to a decrease in the C/N ratio (Table 1).

**TABLE 1:** Contents of organic C and N and C/N ratio of substrate with and without compost for the experiment on *Acacia dealbata* and *Ailanthus altissima*.

	Without compost	With compost	*p*‐Value
*A. dealbata*			
N (%)	0.506 ± 0.001	0.774 ± 0.02	.007**
C (%)	14.243 ± 0.386	15.292 ± 0.466	.158^ns^
C/N	28.143 ± 0.727	19.754 ± 0.181	.007**
*A. altissima*			
N (%)	0.487 ± 0.007	0.647 ± 0.038	.053^*ms*^
C (%)	12.861 ± 0.269	13.694 ± 0.575	.281^ns^
C/N	26.406 ± 0.816	21.215 ± 0.497	.012*

*Note*: The results are those of *t*‐tests: * for *p* ≤ .05, ** for *p* ≤ .01, *** for *p* ≤ .001, *ms* for *p* ≤ .1, and ns for nonsignificant.

The pots were watered once at the beginning of the experiment when one seed was placed in each pot. Depending on the watering modality considered, 20 ml of leaf aqueous extract at 2.5% or 10% of water (control) was added to each pot.

Fifty replicates were made for each treatment for a total of 500 pots of *A. dealbata*: source species (*C. coggygria* and *C. ladanifer*) × extract dose (2,5% and 10%) × soil amendment (without and with compost); and 500 pots of *A. altissima*: source species (*C. coggygria* and *C. albidus*) × extract dose (2.5% and 10%) × soil amendment (without and with compost). Pots were placed randomly into climatic chambers at 25°C, with lighting reproducing a day from 7 AM to 7 PM. Preliminary tests allowed us to determine the optimal relative humidity in the climatic chambers, that is, 85% and 75% for *A. dealbata* and *A. altissima*, respectively.

### 2.3 Seed germination and seedling growth measurements

Seed germination was monitored daily. It was observed when the radicle pierced the seed coat (visible radicle protrusion ≥2 mm, (Dessì et al., 2021)). The final germination percentage was calculated as (number of germinated seeds)/(the number of sown seeds) × 100 (Hashoum et al., 2017). The germination duration is expressed in days and corresponds to the time required for the seed to germinate from the sowing date.

Concerning seedling growth, radicle and hypocotyl lengths (mm) were measured for each individual 8 days after seed germination. After size measurements, the seedlings were freeze‐dried (CHRIST Alpha 1–4 LD_plus_) and weighed to estimate seedling biomass (mg).

### 2.4 Chemical characterization of leaf aqueous extracts by FTIR‐ATR (Fourier‐transformed infrared‐attenuated Total reflection)

Leaf aqueous extracts were freeze‐dried (CHRIST Alpha 1‐4 LD_plus_) and ground using a ball mill (Retch). Powders were deposited onto Specac's Golden Gate™ ATR Accessory of a Thermo Nicolet IS10 spectrometer equipped with a MCT detector, an Ever‐Glo source and a KBr/Ge beam‐splitter. Spectra were acquired between 4000 and 650 cm^−1^, with a 4 cm^−1^ nominal resolution. For each spectrum, 100 scans were co‐added. A background spectrum in air (with the same acquisition conditions as those used for the samples) was acquired before each acquisition. Three spectra were recorded for each sample. OMNIC software (Version 8.1, *Thermo* Scientific, USA) was used to record FTIR‐ATR spectra. The spectral range of the absorption of the carbon dioxide was removed (between 2400 and 1900 cm^−1^) and then a Multiplicative Scatter Correction was applied to remove the slope variation from the spectra caused by scatter and variation of particle size.

### 2.5 Statistical analyses

The relative allelopathic effect (RAE) index was used to determine the intensity of the allelopathic effect on the seed germination velocity and on the seedling growth parameters (Gavinet et al., 2019; Hashoum et al., 2017). RAE is defined as (O − C)/C × 100%, where O is the mean value measured when a target species is exposed to allelopathic compounds and C is the mean value measured for the control. A negative RAE indicates an inhibitory effect of allelopathic compounds, while a positive RAE indicates a stimulatory effect.

Statistical analyses were performed using the R software (RStudio 2022.07.2). We used nonparametric tests due to the non‐normality of the data.

To compare plant species performances in the absence of leaf aqueous extract (i.e., control treatments), a *χ* ^2^ test was used to test for the effect of compost on the germination rate, while a one‐sample Wilcoxon–Mann–Whitney test was used to test for the effect of compost on the germination duration and on the seedling growth parameters (biomass, hypocotyl length, and radicle length).

To evaluate the effect of source species and extract dose, first, one‐sample Wilcoxon‐Mann‐Whitney tests were used to evaluate whether RAE values were significantly different from zero for each combination of source species × extract dose × amendment. Second, one‐sample Wilcoxon–Mann–Whitney tests were used to compare RAE values between high and low extracts for each species × amendment combination.

## 3 RESULTS

### 3.1 Seed germination and seedling growth parameters in absence of allelochemicals

The presence of compost only altered the germination parameters of the two target species (Table 2): the germination duration of both species increased and the germination rate of *Ailanthus altissima* decreased.

**TABLE 2:** Germination and growth traits of the *Acacia dealbata* and *Ailanthus altissima* seedlings with or without compost for the control treatments (i.e. in absence of leaf aqueous extracts).

	Without compost	With compost	Comparison	*p*‐Value
*A. dealbata*				
Germination rate (%)	88	82	*χ* ^2^ = 1.2	.2766^ns^
Germination duration (day)	2.98 ± 1.48	6.68 ± 2.22	W = 611.5	.0082**
Hypocotyl size (mm)	22.60 ± 1.27	24.42 ± 1.81	W = 699.5	.3446^ns^
Radicle size (mm)	76.31 ± 4.40	70.82 ± 5.12	W = 992.5	.0615^ns^
Seedling biomass (mg)	6.16 ± 0.24	5.84 ± 0.25	W = 873.0	.4644^ns^
*A. altissima*				
Germination rate (%)	94	86	*χ* ^2^ = 4.3	.0371*
Germination duration (day)	4.45 ± 0.40	5.16 ± 0.32	W = 705.0	.0082**
Hypocotyl size (mm)	27.43 ± 1.07	25.65 ± 0.92	W = 1103.5	.1120^ns^
Radicle size (mm)	65.48 ± 3.53	61.97 ± 3.11	W = 1015.5	.4106^ns^
Seedling biomass (mg)	14.46 ± 0.51	14.07 ± 0.46	W = 977.0	.6224^ns^

*Note*: Data are mean ± SE. *χ* ^2^ test was used for the comparison of germination rates, while Wilcoxon tests for the comparison of other measured parameters. Test values and associated *p*‐values are indicated. * for *p* ≤ .05, ** for *p* ≤ .01, *** for *p* ≤ .001 and ns for nonsignificant.

### 3.2 Allelopathic interactions in absence of compost

#### 3.2.1 Acacia dealbata

**FIGURE 1:**
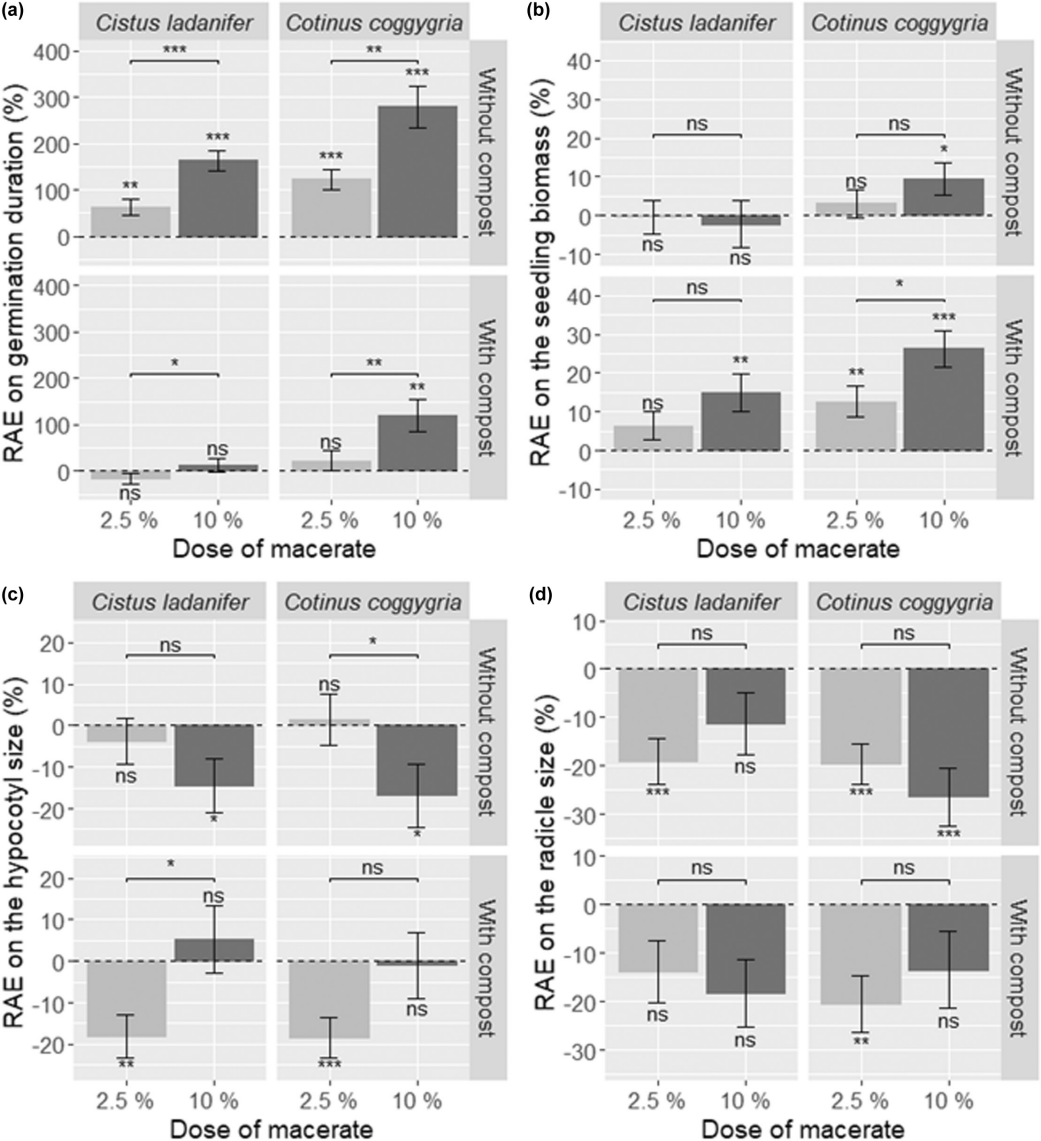
Relative allelopathic effect (RAE) on germination duration (a), biomass (b), hypocotyl size (c), and radicle size (d) of *Acacia dealbata* seeds and seedlings. Each bar represents the mean value ± SE. The results are those of Wilcoxon tests: * for *p* ≤ .05, ** for *p* ≤ .01, *** for *p* ≤ .001, and ns for nonsignificant.

The germination rate of *Acacia dealbata* decreased significantly only in the presence of the high‐dose *C. ladanifer* leaf aqueous extract (Table 3), from 88% to 70%. The germination was slowed in the presence of *C. ladanifer* and *C. coggygria* leaf aqueous extracts at both doses, but this delay in germination was significantly greater in the presence of the high‐dose aqueous extracts (Figure 1a).

**TABLE 3:** Effects of leaf aqueous extracts of *Cistus ladanifer* and *Cotinus coggygria* on the germination rates of *Acacia dealbata*.

Source species	Dose of macerate	Germination rate (%)	*χ* ^2^
Without compost			
*C. ladanifer*	2.5%	86	0.05^ns^
*C. ladanifer*	10%	70	13.7***
*C. coggygria*	2.5%	92	0.4^ns^
*C. coggygria*	10%	80	2.3^ns^
With compost			
*C. ladanifer*	2.5%	90	1.7^ns^
*C. ladanifer*	10%	74	1.7^ns^
*C. coggygria*	2.5%	96	5.7*
*C. coggygria*	10%	74	1.7^ns^

*Note*: The data are compared to the germination rates of controls without compost (88%) and with compost (82%) (Table 2). *χ* ^2^ tests were performed, and proportions that were significantly different from the control are indicated by the respective symbols: * for *p* ≤ .05, ** for *p* ≤ .01, *** for *p* ≤ .001, and ns for nonsignificant.

*A. dealbata* seedling biomass was 9.6% higher in the presence of 10% *C. coggygria* leaf aqueous extract (Figure 1b). The hypocotyl size was 14.5% and 16.9% shorter in the presence of high‐dose leaf aqueous extracts of *C. ladanifer* and *C. coggygria*, respectively (Figure 1c). Finally, the radicle size significantly decreased in the presence of *C. coggygria* leaf aqueous extract at all concentrations (Figure 1d), while only the low dose of *C. ladanifer* leaf aqueous extract negatively affected the radicle size of *A. dealbata* seedlings (Figure 1d).

#### 3.2.2 Ailanthus altissima

**FIGURE 2:**
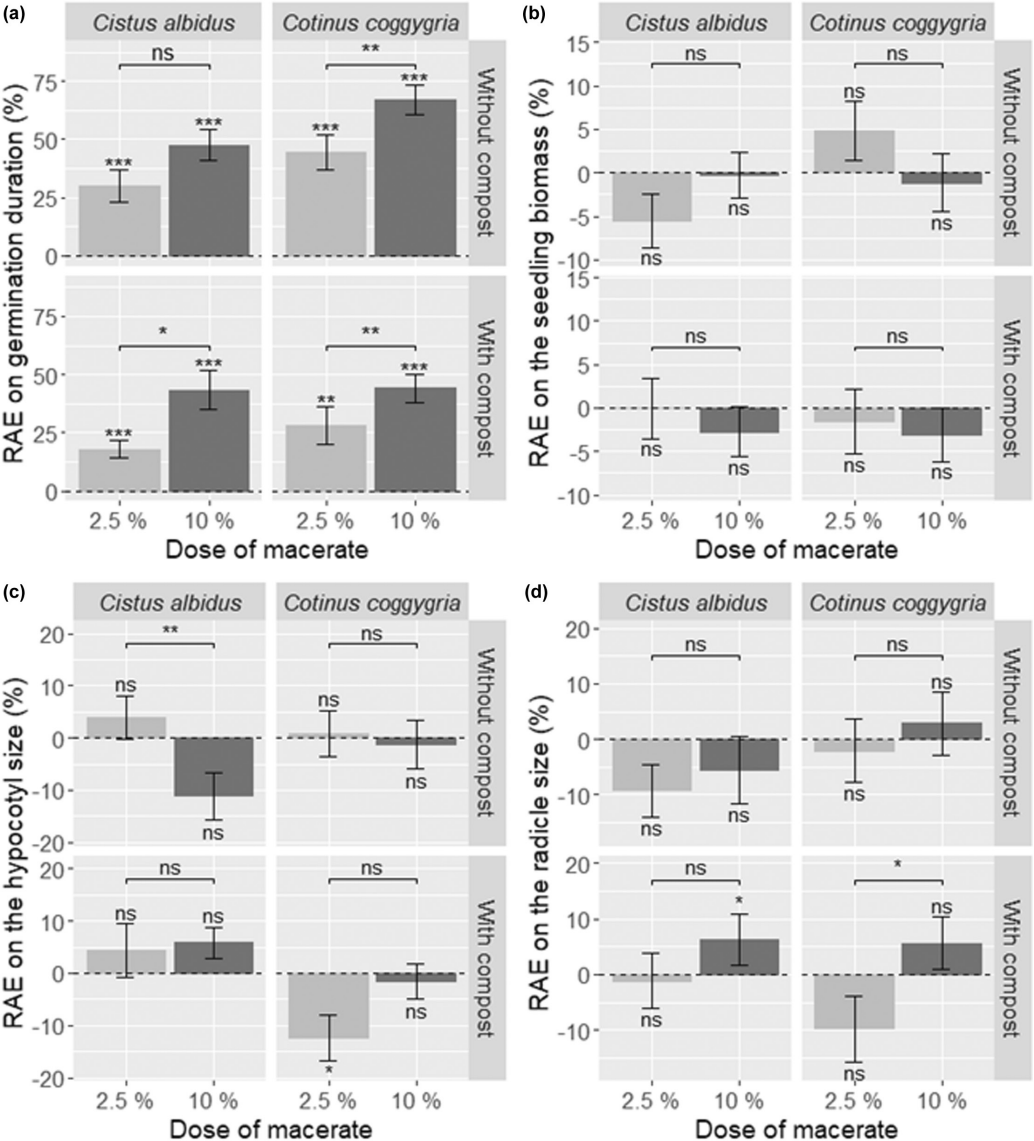
Relative allelopathic effect (RAE) on germination duration (a), biomass (b), hypocotyl size (c) and radicle size (d) of *Ailanthus altissima* seeds and seedlings. Each bar represents the mean value ± SE. The results are those of Wilcoxon tests: * for *p* ≤ .05, ** for *p* ≤ .01, *** for *p* ≤ .001, and ns for nonsignificant.

The germination rate of *A. altissima* was significantly reduced in the presence of leaf aqueous extracts (Table 4), especially in the presence of high‐dose *C. coggygria* leaf aqueous extract (94% vs. 70%). Germination was delayed by the presence of both *C. albidus* and *C. coggygria* leaf aqueous extracts, and this allelopathic effect increased with extract concentration only for *C. coggygria* (Figure 2a).

**TABLE 4:** Effects of leaf aqueous extracts of *Cistus albidus* and *Cotinus coggygria* on the germination rates of *Ailanthus altissima*.

		Germination rate (%)	*χ* ^2^
Without compost			
*C. albidus*	2.5%	82	10.7**
*C. albidus*	10%	86	4.3*
*C. coggygria*	2.5%	84	7.2**
*C. coggygria*	10%	70	46.9***
With compost			
*C. albidus*	2.5%	88	0.04^ns^
*C. albidus*	10%	84	0.04^ns^
*C. coggygria*	2.5%	86	0.00^ns^
*C. coggygria*	10%	90	0.4^ns^

*Note*: The data are compared to the germination rates of controls without (94%) and with compost (86%) (Table 2). *χ* ^2^ tests were performed, and proportions that were significantly different from the control are indicated by the respective symbols: * for *p* ≤ .05, ** for *p* ≤ .01, *** for *p* ≤ .001, and ns for nonsignificant.

*Ailanthus altissima* seedling biomass, hypocotyl size, and radicle size were not affected by the presence of leaf aqueous extracts (Figure 2b–d).

### 3.3 Effects of allelochemicals in presence of compost

#### 3.3.1 Acacia dealbata

In the presence of compost, the germination rate of *A. dealbata* was not affected by *C. ladanifer* leaf aqueous extract but increased with low‐dose *C. coggygria* aqueous extract (from 82 to 96%, Table 3). In contrast, the germination was delayed by 119% in the presence of high‐dose *C. coggygria* aqueous extract (Figure 1a).

The biomass of *A. dealbata* seedlings was increased in the presence of *C. coggygria* leaf aqueous extracts (Figure 1b), with a greater negative effect at high doses, while this increase occurred with *C. ladanifer* aqueous extract only at the high dose (Figure 1b). The hypocotyl size was negatively affected by low‐dose aqueous extracts, whatever the source species considered (Figure 1c). Finally, the radicle size was reduced only in the presence of low‐dose *C. coggygria* leaf aqueous extract (Figure 1d).

#### 3.3.2 Ailanthus altissima

In the presence of compost, the germination rate was not affected by the leaf aqueous extracts of either source species (Table 4). However, the germination of *A. altissima* was delayed by both *C. ladanifer* and *C. coggygria* leaf aqueous extracts (Figure 2a), and this delay increased with increasing extract dose.

In the presence of compost, the biomass of *A. altissima* seedlings was not affected by leaf aqueous extracts (Figure 2b). The hypocotyl size of *A. altissima* was 12% shorter in the presence of low‐dose *C. coggygria* leaf aqueous extract (Figure 2c), whereas the radicle size was 6% higher in the presence of high‐dose *C. albidus* leaf aqueous extract (Figure 2d).

### 3.4 FTIR‐ATR characterization of leaf aqueous extracts

The FTIR‐ATR signatures of *C. coggygria*, *C. albidus*, and *C. ladanifer* leaf aqueous extracts revealed the functional groups of the chemical constituent families extracted in the aqueous phase (Figures S1–S3, respectively). Their FTIR‐ATR profiles showed spectral differences in the region of 1800–650 cm^−1^ between *C. coggygria* and *Cistus* species as well as between *C. albidus* and *C. ladanifer*. The assignment of their spectral bands is reported in Table S1. The spectra of *C. coggygria* and *C. ladanifer*. leaf aqueous extracts showed a characteristic band of acid forms (at 1710 and 1707 cm^−1^, respectively), while this band was not significantly present in the spectral profile of *C. albidus* leaf aqueous extracts. A large band around 1600 cm^−1^ and associated bands between 900 and 750 cm^−1^ were observed in all spectra due to the presence of aromatic compounds such as phenolic compounds, flavonoids and tanins. The intense band around 1030–1040 cm^−1^ confirmed the presence of water‐soluble polysaccharides in these aqueous extracts. A shoulder appeared at 1540 cm^−1^ in the spectra of *C. coggygria* leaf aqueous extracts, characterizing the amine group present in the protein structure.

## 4 DISCUSSION

The aim of this study was to evaluate, under controlled conditions, the allelopathic potential of three native Mediterranean plant species on two invasive plant species. Indeed, leaf aqueous extracts of *Cistus ladanifer*, *Cistus albidus*, and *Cotinus coggygria* showed allelopathic effects on the seed germination and seedling growth of *Acacia dealbata* and *Ailanthus altissima*. However, these effects varied according to the extract dose, the target plant species, and the presence/absence of compost.

### 4.1 Allelochemicals from *Cotinus coggygria* and cistus species inhibit the seed germination of *Acacia dealbata* and *Ailanthus altissima*

According to our first hypothesis, our results pointed out a negative effect of *C. albidus*, *C. ladanifer*, and *C. coggygria* leaf aqueous extracts on seed germination (mainly germination duration) of the two invasive plant species *A. dealbata* and *A. altissima*. This critical life stage is usually affected by allelochemicals (Gallet & Pellissier, 2002). They can, for example, induce a modification of membrane structure and transport receptors or interfere with the cell cycle (Reigosa et al., 2006). Thus, our study can confirm the allelopathic potential of these three Mediterranean plant species recorded in the literature (Chaves et al., 2003, 2016; Chaves & Escudero, 1997; Gavinet et al., 2019; Hashoum et al., 2017, 2021; Robles et al., 1999). In particular, Hashoum et al. (2017) reported, with laboratory experiments, the inhibitory effect of *C. coggygria* leaf aqueous extract on seed germination of *Linum perenne* and *Festuca ovina*, and Chaves and Escudero (1997) highlighted, likewise, the inhibitory effect of *C. ladanifer* leaf aqueous extract on seed germination of *Cynodon dactylon* and *Rumex crispus*. By reducing the germination rate or delaying seed germination, the Mediterranean plant species studied could limit the early establishment of the two invasive plant species. Indeed, in Mediterranean ecosystems, germination takes place during the period when temperature and humidity are suitable for the growth of young seedlings, that is, before the summer drought period (Gulias et al., 2004). A delay in germination caused by allelochemicals could lead to a decrease in the number of individuals of *A. altissima* and *A. dealbata* by reducing the favorable period for seedling establishment (Chaves & Escudero, 1997). However, these laboratory bioassays allow us to identify and screen for allelopathic potentialities, but due to their slow release and low concentration, it can be difficult to relate them directly to allelopathic interactions occurring on the field (Fernandez et al., 2013). Following our results, *in situ* experiments could help to investigate a new nature‐based solution.

According to our second hypothesis, the allelopathic effects were negative and generally increased with increasing dose extract. Some previous studies have shown a stimulatory effect on seed germination that increased with dose extract or occurred only at low dose extract (Fernandez et al., 2013; Liu & Lovett, 1990; Santonja et al., 2018). For example, Fernandez et al. (2013) showed that high‐dose *Pinus halepensis* needle aqueous extracts induced a slowdown in the germination velocity of several herbaceous species (*Arabis hirsuta*, *Daucus carota*, *Linum strictum*, *Reichardia picroides*, *Salvia verbenaca*, *Sedum sediforme*, and *Trifolium stellatum*), while an increase in the germination velocity of two other species was observed at low‐dose (*Helichrysum stoechas* and *Tanacetum corymbosum*). In the present study, we did not observe a stimulation of germination, but a decrease in germination rate or a delay in germination, which was greater in the presence of 10% leaf aqueous extracts. These results confirm those of Hashoum et al. (2017), who also observed a greater inhibition in the presence of *C. coggygria* leaf aqueous extracts at high dose than at low dose.

### 4.2 Allelochemicals from *Cotinus coggygria* and cistus species differentially affect the seedling growth of *Acacia dealbata* and *Ailanthus altissima*

The radicle growth of *A. dealbata* seedlings was negatively affected by *C. coggygria* and *C. ladanifer* leaf aqueous extracts, but not hypocotyl growth or seedling biomass. Several studies have demonstrated that the root part of the target plants is more sensitive to allelochemicals than the aerial part (Gatti et al., 2010; Zhang & Fu, 2010). This sensitivity could be related to the absorption role of the roots, which implies direct contact with the allelochemicals present in the soil (Turk & Tawaha, 2003). Furthermore, Šoln et al. (2022) showed that, in allelopathic interactions between plants, the structure and activity of the apical meristem of the target species roots are altered, affecting root growth and water uptake. These inhibitory effects on root growth weaken the water nutrition of the target plant (Robles et al., 1999). Under the Mediterranean climate, the successful establishment of a plant species largely depends on a well‐developed root system for efficient resource acquisition (Green et al., 2005), especially when water uptake is a limiting factor. Inhibiting root development can significantly reduce the performance of the target species, making them less tolerant to drought (Herranz et al., 2006). Effects on seedling growth can alter the competitiveness of the invasive plant species for access to resources, development, and survival.

Contrary to *A. dealbata*, the growth of *A. altissima* was not affected by the presence of *C. coggygria* leaf aqueous extracts. This result highlights that allelopathic interactions depend on the target species considered and the ecological context (Callaway & Aschehoug, 2000; Hierro & Callaway, 2003). This may be due to differences in seed size, seed coat permeability, differential uptake, or metabolism (Haugland & Brandsaeter, 1996). Consequently, our study points out that *A. dealbata* appears to be more sensitive to allelochemicals of native Mediterranean plant species than *A. altissima*, since both germination and growth stages are inhibited.

Our results also highlight that the effect of leaf aqueous extracts on the different parameters studied (germination and growth) depends on the source plant species. This can be explained by differences in the chemical composition of these leaf aqueous extracts, explored from their spectral signatures recorded in the mid‐infrared (MIR) region (Figures S1–S3). The spectral signatures of *C. coggygria*, *C. ladanifer*, and *C. albidus* aqueous extracts revealed the functional groups of the chemical constituents extracted in the aqueous phase (Table S1). Different spectral bands of varying intensity were observed, suggesting that the extracted chemical compounds belonged to different families of compounds and that their concentration varied from one plant to another, depending on the concentration of the aqueous extract. Their MIR fingerprints showed, among others, specific bands revealing the presence of phenolic compounds, often assumed to be responsible for the allelopathic behavior of plants (Fernandez et al., 2016; Gavinet et al., 2019; Santonja et al., 2018). The latter could be assigned to signals from phenolic acids, flavonoids (anthocyanin, flavonol, flavanol, flavanone, and flavonone), or tannins (condensed and hydrolysable) that need to be identified and quantified by coupled chromatographic methods.

### 4.3 Presence of compost greatly diminish the allelopathic interactions between native and invasive plant species

According to our third hypothesis, the negative effect of *C. coggygria* and *Cistus* species leaf aqueous extracts on the germination parameters of *A. dealbata* and *A. altissima* was less significant in the presence of soil amendment. Similarly, the negative impact on the radicle growth of *A. dealbata* was reduced in the presence of soil amendment. Therefore, this finding points out that the addition of compost probably acts as a buffer and limits the effect of allelochemical compounds. First, it can be assumed that allelopathic compounds are adsorbed on the organic matter of the compost (Rice, 1984). Second, soil physicochemical and mechanical properties (e.g. permeability or water retention) may play a role in the diffusion of allelopathic compounds (Parepa & Bossdorf, 2016), and the presence of nutrient resources may counteract the action of allelochemicals (Einhellig, 1987; Lehman & Rice, 1972). In addition, our experiment does not allow us to identify whether the allelopathy pathway acts directly on target plant species or indirectly through effects on soil biota. Indeed, soil microbial communities mediate allelopathic interactions (Fernandez et al., 2013; Gavinet et al., 2019) and can transform allelochemicals into less or more toxic compounds (Inderjit, 2005; Lankau, 2010; Li et al., 2017). Further experiments would allow us to explore the effect of the amendment on microorganisms and their control on plant–plant allelopathic interactions. By limiting the inhibitory effect of native Mediterranean plants on the germination of invasive alien plants, the addition of compost does not appear to be a method to consider in the control of invasive alien plants. In fact, even if the addition of compost could aid in the establishment of native plants on railways, invasive alien plants could also benefit from it, as described above.

## 5 CONCLUSION

This study provides new insights into the use of allelopathy as a nature‐based solution to control IAS in the Mediterranean region and should be taken into account to limit their proliferation along railway lines. Indeed, thanks to their allelopathic potentialities, the installation of native Mediterranean plants (*Cistus albidus*, *C. ladanifer*, and *Cotinus coggygria*) could limit the spread of *Ailanthus altissima* and *Acacia dealbata*. The delay in seed germination induced by allelochemicals produced by native plants can lead to a decrease in the individual recruitment of invasive alien plants. In addition, a reduction in seedling growth can alter the competitiveness of invasive species for resource access. However, the addition of compost limits the inhibitory effect of native Mediterranean plants on the germination of invasive alien plants, suggesting that an efficient method to manage invasive alien plants does not include the addition of compost. Complementary studies, exploring, for example, interactions with soil organisms or in‐situ experiments, would provide further insight.

## AUTHOR CONTRIBUTIONS

**Solène Brasseur:** Conceptualization (equal); data curation (equal); formal analysis (equal); investigation (equal); methodology (equal); software (equal); visualization (equal); writing – original draft (equal); writing – review and editing (equal). **Mathieu Santonja:** Conceptualization (equal); formal analysis (equal); methodology (equal); software (equal); writing – original draft (equal); writing – review and editing (equal). **Catherine Rébufa:** Formal analysis (equal); methodology (equal); software (equal); visualization (equal); writing – review and editing (equal). **Laurence Affre:** Conceptualization (equal); funding acquisition (equal); writing – review and editing (equal). **Sylvie Dupouyet:** Conceptualization (equal); data curation (equal); investigation (equal); methodology (equal); resources (equal). **Estelle Dumas:** Writing – review and editing (equal). **Thierry Tatoni:** Funding acquisition (equal); project administration (equal); writing – review and editing (equal). **Anne‐Marie Farnet Da Silva:** Conceptualization (equal); funding acquisition (equal); supervision (equal); writing – original draft (equal); writing – review and editing (equal). **Anne Bousquet‐Mélou:** Conceptualization (equal); data curation (equal); funding acquisition (equal); investigation (equal); methodology (equal); supervision (equal); writing – original draft (equal); writing – review and editing (equal).

## CONFLICT OF INTEREST STATEMENT

The authors declare that they have no known competing financial interests or personal relationships that could have appeared to influence the work reported in this paper.

## Supporting information


Appendix S1.

## Data Availability

The data supporting this article are available on demand.
