# Supplementary material for: Can allelopathic potentialities of Mediterranean plant species reduce the spread of invasive plant species, Acacia dealbata and Ailanthus altissima?
Source: Ecol Evol. 2024 Jun 25;14(6):e11499. doi: 10.1002/ece3.11499 (PMC11199123; doi:10.1002/ece3.11499)
Supplement: Supplementary file 1 — Appendix S1. [file ECE3-14-e11499-s001.docx]

**Supplementary material**

***Supplementary Figure S1.*** FTIR-ATR corrected spectra of *Cotinus coggygria* leaf aqueous extracts.


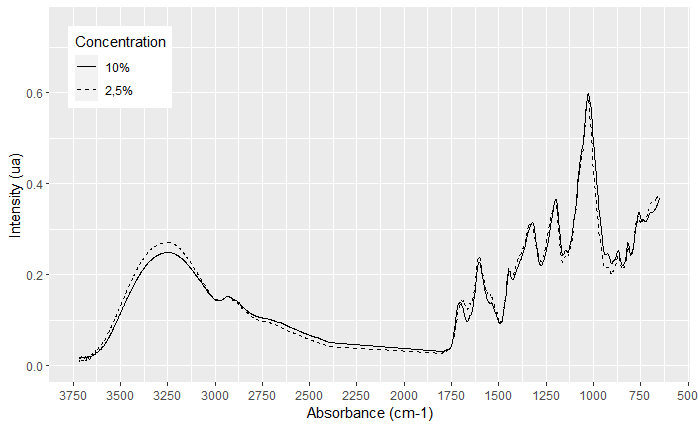


761 cm^-1^

818 cm^-1^

868 cm^-1^

1200 cm^-1^

1325 cm^-1^

1446 cm^-1^

1540 cm^-1^

1600 cm^-1^

1710 cm^-1^

2931 cm^-1^

3250 cm^-1^

1030 cm^-1^

***Supplementary Figure S2.*** FTIR-ATR corrected spectra of *Cistus albidus* leaf aqueous extracts.


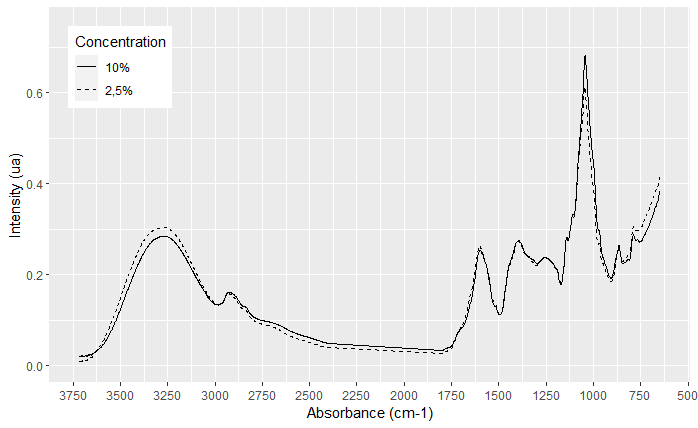


3280 cm^-1^


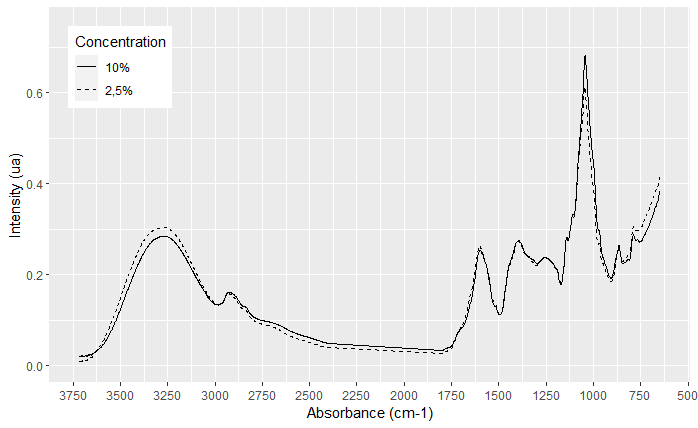


2923 cm^-1^

2837 cm^-1^

1600 cm^-1^

1394 cm^-1^

1250 cm^-1^

864 cm^-1^

788 cm^-1^

1043 cm^-1^

***Supplementary Figure S3.*** FTIR-ATR corrected spectra of *Cistus ladanifer* leaf aqueous extracts.


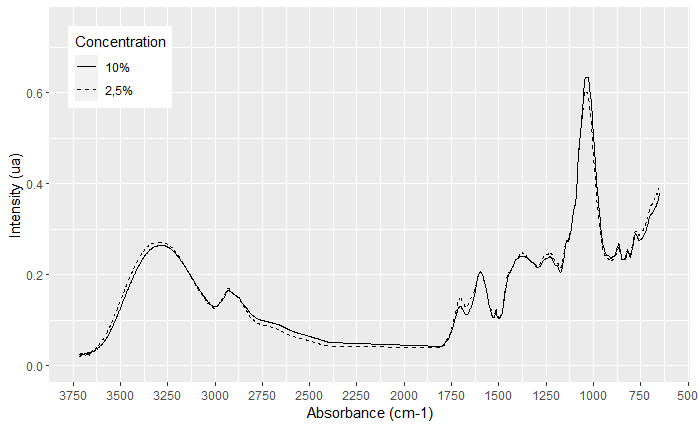


3296 cm^-1^

2932 cm^-1^

1707 cm^-1^

1600 cm^-1^

1516 cm^-1^

1375 cm^-1^

1223 cm^-1^

1040 cm^-1^

864 cm^-1^

818 cm^-1^

771 cm^-1^

***Supplementary Table S1.*** Absorption band assignation.

|  | *Cistus ladanifer* | *Cistus albidus* | *Cotinus coggygria* |
| --- | --- | --- | --- |
| ν(O-H, N-H) | 3296 | 3280 | 3250 |
| ν_as_ (C-H in CH_2_, CH_3_) | 2932 | 2923 | 2931 |
| ν_s_ (C-H in CH_3_) |  | 2837 |  |
| ν (C=O acid) | 1707 |  | 1710 |
| ν (C=C aromatic) | 1600 | 1600 | 1600 |
| ν (C-N amide II) |  |  | 1540 |
| ν (C-C aromatic) | 1516 |  |  |
| ν (C-C aromatic), δ_as_ (C-H in CH_2_, CH_3_) |  |  | 1446 |
| δs (C-H in CH_2_, CH_3_) | 1375 | 1394 |  |
| ν (C-O alcohols, ether, ester), ν (C-C) |  | 1250 | 1325 |
| δ (C-OH alcohol) | 1223 |  | 1200 |
| ν (C-O-C glycosydic), ν (C-C), ν (C-O) | 1040 | 1043 | 1030 |
| γ (C-H aromatic) | 864 | 864 | 868 |
| γ (C-H aromatic) | 818 |  | 818 |
| γ (C-H aromatic) | 771 | 788 | 761 |

*ν:*  stretching, *δ:* in plane bending and *γ:* out-of-plane deformation
